# Signal Transduction during Metabolic and Inflammatory Reprogramming in Pulmonary Vascular Remodeling

**DOI:** 10.3390/ijms23052410

**Published:** 2022-02-22

**Authors:** Marta T. Gomes, Yang Bai, Simone R. Potje, Lu Zhang, Angelia D. Lockett, Roberto F. Machado

**Affiliations:** 1 Division of Pulmonary, Critical Care, Sleep and Occupational Medicine, School of Medicine, Indiana University, Indianapolis, IN 46202, USA; yb2@iu.edu (Y.B.); simonepotje@hotmail.com (S.R.P.); anlocket@iu.edu (A.D.L.); 2 Department of Clinical Pharmacology, School of Pharmacy, China Medical University, Shenyang 110122, China; 3 Department of Biological Science, Minas Gerais State University (UEMG), Passos 37900-106, Brazil; 4 Department of Ion Channel Pharmacology, School of Pharmacy, China Medical University, Shenyang 110122, China; lzhang95@cmu.edu.cn

**Keywords:** pulmonary arterial hypertension, vascular remodeling, signaling pathways, metabolism, inflammation

## Abstract

Pulmonary arterial hypertension (PAH) is a progressive disease characterized by (mal)adaptive remodeling of the pulmonary vasculature, which is associated with inflammation, fibrosis, thrombosis, and neovascularization. Vascular remodeling in PAH is associated with cellular metabolic and inflammatory reprogramming that induce profound endothelial and smooth muscle cell phenotypic changes. Multiple signaling pathways and regulatory loops act on metabolic and inflammatory mediators which influence cellular behavior and trigger pulmonary vascular remodeling in vivo. This review discusses the role of bioenergetic and inflammatory impairments in PAH development.

## 1. Introduction

Pulmonary hypertension (PH) is a progressive disease characterized by increased pulmonary vascular resistance (PVR) leading to right heart hypertrophy and ultimately death due to right heart failure [1]. Hemodynamically, PH is defined as a mean pulmonary artery pressure (mPAP) of >20 mmHg, pulmonary artery wedge pressure ≤ 15 mmHg and PVR ≥ 3.0 Wood units [2]. Recently, clinical aspects associated with pathophysiology, etiologies, clinical presentation, hemodynamic characteristics, and therapeutic management conditions were re-evaluated and revised during the 6th World Symposium on Pulmonary Hypertension (WSPH) [2]. PH is classified in 5 groups: (1) pulmonary arterial hypertension (PAH), which comprises diverse diseases that cause similar pathological changes within the pulmonary vasculature, that will be described below; (2) left heart diseases like systolic or diastolic heart failure and left sided valvular diseases; (3) lung parenchyma or hypoxia related diseases; (4) chronic thromboembolic pulmonary hypertension (CTEPH) and other pulmonary obstructive processes; (5) diseases with multifactorial mechanisms or unclear mechanisms, including hematological disorders and, systemic and metabolic disorders [2,3].

PAH is characterized by increases in PVR that are mostly due to (mal)adaptive remodeling of the pulmonary vasculature, which is associated with inflammation, fibrosis, thrombotic lesions, medial hypertrophy, and intimal proliferation. Plexiform lesions and remodeling of the small pre-capillary pulmonary arterioles constitute the hallmarks of the disease [4].

Due to its complexity and being constituted by several associated clinical disorders, PAH is subdivided in: idiopathic PAH (group 1.1); heritable PAH (group 1.2); drug- and toxin-induced PAH (group 1.3); PAH associated with various conditions including connective tissue diseases, such as HIV infection, portal hypertension, and congenital heart disease (group 1.4); PAH in long-term responders to calcium channel blockers (group 1.5); PAH with venous/capillary involvement (group 1.6); and persistent PH of the newborn (group 1.7) [5]. Genetic mutations in Bone Morphogenetic Protein Receptor Type 2 (BMPR2) remain the most common cause of PAH, account for ~80% of hereditary (HPAH) and ~20% idiopathic PAH (IPAH) [6]. Besides that, other hereditary causes of PAH involve TGF-β superfamily genes including ALK1/ACVRL1 (a heterodimeric partner of BMPR2), BMP9 (a BMPR2 family member), ENG (a coreceptor for BMPR2 signaling), and SMAD1, 4, and 9 (downstream BMP signaling molecules) have been linked to both HPAH and IPAH [7]. Recently, two other genes have been associated: CAV1 (involved in BMPR2 membrane localization and signaling) [8] and KCNK3 (a potassium channel that regulates resting membrane potential) [2,9].

Although in the last few decades there has been progress in understanding of PAH, cure of the disease remains an unmet challenge, as this group is the most aggressive form of PH, with poor survival and limited treatment options. According to Registry to Evaluate Early and Long-term PAH disease management study (REVEAL) there is an estimated five-year survival of 57% from the time of diagnostic right heart catheterization [2]. Thus, it is extremely necessary to understand the cellular and molecular mechanisms underlying this disease, in order to identify new intervention mechanisms.

In this review, we will summarize current data on the mechanisms that control metabolic and inflammatory events in endothelial cells (ECs) and smooth muscle cells (SMCs) as essential pathways leading to pulmonary vascular remodeling in PAH.

## 2. Metabolic Changes Related to Vascular Remodeling

Although the mechanisms involved with dysfunction in energy metabolism are not fully understood in PAH, it is known that metabolic shifts mediating abnormalities in PAH PAECs and PASMCs are directly linked to an imbalance between glycolysis, glucose oxidation, and fatty acid oxidation [10,11,12,13,14]. In cells, during energy substrate metabolism, glucose is metabolized to pyruvate in the cytosol and can follow different paths: (1) in mitochondria, pyruvate undergoes oxidative phosphorylation (OXPHOS) entering the tricarboxylic acid cycle (TCA), where it will generate 32–36 ATPs per glucose molecule, or (2) it can be converted into lactate (anaerobic glycolysis), which will generate only 2 ATPs per glucose molecule. 

### 2.1. Glycolysis and the Warburg Effect

Despite the abundance of oxygen in the blood and the direct and unlimited access of blood vessel-lining ECs to oxygen, ECs prefer to use aerobic glycolysis as the major metabolic pathway to generate energy, which might be beneficial when ECs sprout into avascular tissue. This effect is known as the Warburg phenomenon, where cells exhibit a metabolic change in which mitochondrial glucose oxidation is suppressed [15]. Uebelhoer et al. [15] discussed in their paper some advantages for ECs to preferentially use glycolysis over OXPHOS, as a low-oxidative metabolism limits the formation of reactive oxygen species (ROS) and potential ROS-mediated damage. In addition, glycolysis can generate ATP much more rapidly, facilitating adaptation to quickly changing energy-demands of proliferating/migrating cells and enabling more oxygen availability for perivascular cells.

In similar fashion to cancer, proliferating cells in PAH switch their metabolism to glycolysis and that has been associated with vascular expansion. For example, the endothelium doubles glycolytic rates when switching from quiescence to an angiogenic phenotype [16]. Supporting this notion, PDG-PET, which which uses ^18^F-fluorodeoxyglucose (^18^FDG), a glucose analogue, as a radiotracer for positron emission tomography (PET) has been applied to monitor the pulmonary vasculature during PAH progression. Studies observed that ^18^FDG uptake is increased in the lungs of patients with idiopathic PAH (PAH) [12,17] reflecting a shift towards glycolytic metabolism. Some analyses in IPAH lungs, however, concluded that ^18^FDG uptake is not homogeneous. In some patients with IPAH ^18^FDG uptake was 3-fold that of control group, whereas others were in the range of controls, suggesting that this variability may be due to extensive morphological heterogeneity within individual lungs and between lungs of patients with IPAH [18]. In animal models, this technique detected an increase of glucose transporter 1 (GLUT1) mRNA, induced by HIF 1α in both endothelium and PASMCs, but not in airway cells or macrophages in rates with MCT induced PH [12]. Based on this information, it is suggested that ^18^FDG PET imaging may be a useful clinical tool to assess the subpopulation of IPAH patients with increased pulmonary glucose metabolism, making it possible to explore the efficacy and dose-response relationships of new treatments targeting PAH [18].

Undoubtedly, alterations in the function of different metabolic enzymes in PAH can be considered potential biomarkers for the diagnosis and prognosis of the disease. For instance, hexokinase-1 has also been identified to be up-regulated in early passage PASMCs derived from MCT animals, indicating metabolic shift in these cells [12]. Hexokinase binds to the voltage-dependent anion channel (VDAC) in the mitochondrial membrane, preventing the release of cytochrome C, thus contributing to the inhibition of apoptosis and promoting cell proliferation [19]. The enzyme PFKBF3, which catalyzes the conversion of fructose 1,6-bisphosphate (F1,6P2) to fructose 2,6-bisphosphate (F2,6P2) in vascular cells, also participates in glycolysis-associated angiogenesis [16,20] and upregulation of PFKFB3 mediates cell proliferation and collagen synthesis in PASMCs [21]. Besides that, the increase in PFKFB3 promotes an immunological response resulting in inflammasome activation [22]. The accumulated pyruvate, generated by PFKFB3 enhances the ability to inhibit prolyl hydroxylase domain proteins, reduces the degradation of hypoxia-inducible factors 1α (HIF-1α) in cells, promotes the expression of IL-1β, CXCL12 and platelet-derived growth factor B (PDGFB), and ultimately leads to PASMC abnormal proliferation and migration [22]. 

Enolase (ENO), the ninth enzyme of glycolysis, which catalyzes the dehydration of 2-phospho-D-glycerate (2-PG) to phosphoenolpyruvate (PEP), is elevated in PAH PASMCs but not in PAECs and fibroblasts or in IPAH suggesting potentially different pathogenetic mechanisms between IPAH and HPAH [23]. Interestingly, ENO antibodies were found in the serum of systemic sclerosis patients with PAH. These antibodies induce VSMCs contraction indicating that ENO1 is as possible regulator metabolic reprogramming in PAH associated with scleroderma [24,25]. Another abnormality occurs in the last step of glycolysis, the conversion of phosphoenolpyruvate to pyruvate and ATP by pyruvate kinase (PK). Some reports observed an increase of pyruvate kinase muscle 2 (PKM2) in PAECs and blood outgrowth ECs (BOECs) of PAH patients and in the Sugen/ hypoxia model, while the PKM1 did not have its activity altered, inferring that polypyrimidine tract binding protein 1 (PTBP1) could be responsible for the selective splicing of PKM2/PKM1 isoform [26,27]. It is important to mention that PKM2 transcription is regulated by HIF1α through its binding to HIF response elements promoter [28]. When the dimeric form of PKM2 is translocated into the nucleus, it phosphorylates nuclear proteins such as STAT3 and β-catenin [29,30,31]. It is known that STAT3 is the main member of the STAT family associated with cardiovascular diseases [32,33,34], and STAT3 activation in PAECs changes proliferative and survival phenotypes [35,36,37].

Increases in PTBP1 expression have been described in cancer cells and are correlated with decreases in the production of the small non-coding RNA miRNA-124, suggesting that in PAH, miR-124/PTBP1/PKM2 promotes glycolysis, increases cell proliferation, and causes apoptosis resistance [38]. In this respect, genetic manipulation by miR124 mimic or PTBP1-silencing regulated the splicing ratios in the pulmonary artery (PA) adventitia which harbors activated fibroblasts (PH-Fibs), preventing cell proliferation, and controlling metabolic derangements [39]. Noteworthy, miRNAs have been associated with the post-transcriptional regulation of genes that lead to proliferation, migration, and pro-inflammatory cytokines, such as IL-6, CCL2/monocyte chemoattractant protein 1 (MCP-1), CCL12/stromal cell derived factor 1, and IL-1β in SMCs, PAECs and fibroblasts in PAH [40,41,42]. For instance, PTBP1 acts in SMC and EC communication, rescues the activation of chromatin remodeling genes by BMPR2, such as Notch1, FOXO3, p21 and p27, which contributes to endothelial regeneration and homeostasis in cells exposed to environmental damage [43]. 

Although increases in glycolysis and lactate levels are well established in PAH [10,11,44,45] and that some studies demonstrated that lactate is a potent activator of proinflammatory genes in macrophages, which stimulates PASMC, PAEC and fibroblast growth [46,47]. Zhao and cols [11] using metabolomics and microarray data, did not detect glycolytic shift or significant changes in lactate levels (characteristic of the Warburg effect) in the lungs of patients with advanced PAH. Furthermore, high levels of glucose, sorbitol and fructose are observed in the lungs of patients with PAH. Possibly, in late-stage PAH, the sorbitol pathway uses excess glucose and, consequently, the potential formation of glycation products can generate free radicals which trigger tissue damage and its association with the PH phenotype. Another possible destination for the glucose consumption suggested by these authors is the pentose phosphate pathway (PPP) [11]. 

### 2.2. Pentose Phosphate Pathway

Although the PPP interfaces with multiple metabolic pathways, little is known about its role in PAH. The PPP is critical because it not only generates pentose phosphates to supply nucleic acid synthesis, but also provides NADPH, which is required for both the synthesis of fatty acids and cell survival under stress conditions. In this context, generation of isocitrate dehydrogenase 1 and 2 (IDH) through increased NADPH activity in PAs and lungs of PAH patients (idiopathic and heritable) has been observed [48,49]. Importantly, NADPH provides a substrate for NADPH oxidase (NOX) to generate ROS. Usually, free NADPH is reduced by glutathione (GSH) or thioredoxin (TRX) redutases–GSH/TRKs- as an antioxidant resource. Under pathological conditions, when metabolic pathways are altered (glycolysis, PPP, and suppression of mitochondrial respiration), vascular NOXs (especially NOX4 and NOX2) have an impact on cell proliferation, apoptosis resistance, as well as activation of cytokines and chemokines that favor the recruitment of immune cells causing vascular injury [50,51]. 

Studies have proposed that BMPR2 mutation stimulates PPP upregulation, which is driven by downstream ribose-5-phosphate isomerase upregulation that overcomes decreases in glucose-6-phosphate dehydrogenase (G6PD) expression and directly enhances nucleotide synthesis and salvage [48]. Alternatively, molecular mechanisms that stimulate G6PD expression and activity in hypoxic and/or endothelin-1 suppress contractile protein expression in cells and promote PASMC, EC and fibroblast proliferation [50,52] which contributes to pulmonary vascular remodeling. G6PD inhibition stimulated by protein kinase G1 (PKG1α) signaling triggered several pathways, such as (1) increases in contractile protein expression [53], (2) reduction in TNFα activation [54], contributing to reduction of pro-inflammatory signals in PA in hypoxia, (3) stimulation of apoptosis in PASMC and other cells through signaling that induces calcium release mediated by IP3 [50]. 

### 2.3. Krebs Cycle

Another disruption associated with PAH is the conversion of pyruvate into acetyl coenzyme A, which inhibits the Krebs cycle. The Krebs cycle is a sequence of reactions that occur within the mitochondria of eukaryotic cells through the release of stored energy by oxidation of acetyl-CoA derived from carbohydrates, lipids, and proteins, which is used as a synthesis of fatty acids, steroids, cholesterol, and amino acids for protein construction and the purines and pyrimidines used in DNA synthesis.

In PAH, mitochondria are the focus of several metabolic changes that result in the dysregulation of several enzymes involved in fatty acid metabolism. For example, SLC25A1, a mitochondrial citrate carrier, which exports citrate from mitochondria to the cytoplasm, has an altered export pathway in PAH. Lower levels of SLC25A1 were shown in PAH PAECs, suggesting that the concentration of mitochondrial citrate may be higher than cytosolic citrate in these cells [14]. Furthermore, citrate increases were shown in PAH lungs [11], and isocitrate and cis-aconitate are also increased in PAH plasma [14]. Notably, these metabolic changes lead to mitochondrial dysfunction that favors cells proliferation and contributes to vascular remodeling in PH.

Pathological activation of PDKs leads to phosphorylation and inhibition of pyruvate dehydrogenase (PDH) resulting in inhibition of acetyl CoA production, reduced electron flux and ROS generation in the mitochondria that is associated with vasoconstriction [12,44,45]. It is suggested that in PAH, the “pseudohypoxic” cellular environment created activates HIF, which mediates several metabolic adaptations. Once HIF is activated, it transactivates two inhibitory PDK isoforms, PDK1 and PDK3 [12,30,55]. Hence, potential mechanisms of PDH inhibition have been extensively studied in PAH. For example, the lungs of patients with PAH have reduced expression of mitochondrial deacetylase SIRT3 and SIRT3 deficiency in mice promotes pulmonary hypertension [56] in a mechanism that involves inhibition of PDH function [45]. 

Besides that, mitochondria from pulmonary arteries and plexiform lesions in patients with PAH are deficient in complex I and superoxide dismutase 2 (SOD2) expression [44]. Studies using genomic bisulfite sequencing demonstrated selective hypermethylation of a CpG island in an enhancer region of intron 2 and another in the promoter, suggesting epigenetic dysregulation of SOD2 possibly associated with upregulation of DNA methyltransferases 1 and 3B [57]. Notably, the restoration of both SOD2 expression and the proliferation-to-apoptosis ratio were reversed by the DNA methyltransferase inhibitor 5-aza-2’-deoxycytidine, suggesting that differential methylation occurs selectively in pulmonary arteries versus aortic SMCs [57]. 

### 2.4. Randle Cycle

The Randle cycle is characterized by competition between glucose and fatty acids for oxidation. In some organs, like the heart, fatty acid oxidation is the major source of ATP production, while glucose metabolism is the secondary source. During the Randle cycle, the production of citrate occurs through fatty acid oxidation, where citrate inhibits phosphofructokinase, causing accumulation of glucose-6-phosphate as a result of hexokinase inhibition which ultimately decreases the production of pyruvate. Randle cycle also involves inhibition of pyruvate dehydrogenase generated during fatty acid oxidation. This metabolic shift occurs in PAH and likely contributes to pathogenesis both in the heart and in the pulmonary vasculature [58,59]. Likewise, in an animal model of PH, inhibition of fatty acid oxidation by blocking the enzyme malonylcoenzyme A decarboxylase (MDC) resulted in attenuation of the disease [60]. 

### 2.5. Glutamine Metabolism

During the production of energy, glutamine is one of the metabolite fuels that can be hydrolyzed to glutamate and subsequently converted to α-ketoglutarate (α-KG) in mitochondria. α-KG can enter the Krebs’ cycle and replenish metabolic intermediates thereby supporting the biosynthetic demands for NADPH and fatty acids. This replenishment of the metabolic intermediates is called anapleurosis, which favors cell growth through the production of ATP. Regarding the metabolic flux, aerobic glycolysis and glutaminolysis are similar pathways. For example, glutamine transporters (SLC1A5 and SLC7A5) [61] are upregulated during glutaminolysis in a similar fashion to GLUT1 transporter in aerobic glycolysis [59,62]. In glutaminolysis, lactate is generated by malic enzyme (Me) [63] and Me2 exerts an important role in glutamate metabolism [64] associated to PAH [48]. 

Alterations in glutamine metabolism have been found in the pulmonary vasculature of patients with PAH [58]. Increases in glutamine metabolism was demonstrated in pulmonary microvascular ECs from BMPR2-mutant mice [65]. Furthermore, upregulation of the glutamine transporter, SLC1A5, was observed in the hypertrophic right ventricle of PAH patients and in the right ventricle of the MCT rat model of PH [66,67]. It is suggested that like in cancer cells, pulmonary vascular cells in PAH use glutamate, through glutamine hydrolysis in an anapleurotic reaction to generate α-ketoglutarate for the tricarboxylic acid cycle [58,68] for the biosynthetic demands for nicotinamide adenine dinucleotide phosphate (NADPH) and fatty acids during rapid cell growth. In concordance, a wide range of glutamate receptors (NMDARs but also AMPA receptors and metabotropic glutamate receptors) expressed in vascular cells could mediate crucial roles in the function and development of the vascular system, such as regulating endothelial barrier permeability and angiogenesis [69].

Data suggest that the increase in glutaminolysis seen in PAH is a result of the vascular extracellular matrix (ECM) stiffness and the activation of transcriptional coactivators YAP/TAZ in response to a metabolic adaption needed to maintain the proliferative state of lung cells [68]. Furthermore, repurposing of the YAP inhibitor verteporfin and CB-839 (GLS-1 inhibitor) for treatment of PH, either separately or together with possible optimization for tissue-specific delivery, is a potential new therapeutic opportunity for this disease [68].

## 3. Mediators That Alter Metabolic Function in PAH

### 3.1. Hypoxia-Inducible Factor

The most important transcription factors related to hypoxic responses are the hypoxia-inducible factors 1 and 2 (HIF1 and HIF2) [70]. Under normoxia, HIF activity is regulated through the oxygen sensor prolyl-4- hydroxylase domain-containing enzymes (PHDs) that use oxygen to hydroxylate specific proline residues of HIF-α. In this situation, HIF-α bind to von Hippel-Lindau (VHL) ubiquitin E3 ligase, leading to ubiquitination and its subsequent degradation by the proteasome [71,72,73]. Therefore, under low concentration of cellular oxygen, HIF-α subunit is activated and translocated to the nucleus where upon dimerization with the HIF-β subunit forms the heterodimeric complex, which binds to the specific DNA binding regions (hypoxia-responsive elements, or HREs) resulting in transcription of its target genes [74].

Several processes that trigger vasoconstriction and vascular remodeling are associated with hypoxia, which makes HIF an essential element in these responses. In cancer cells, HIF promotes numerous roles related to cell proliferation and metabolism. Likewise, the involvement of HIF in PAH pathogenesis is also extensively studied [71,75,76,77].

In PAH, HIF acts on different signaling pathways, where it is suggested that HIF-1α is mostly expressed in PASMCs, while HIF-2α and HIF-3α are expressed in PAECs, and pulmonary fibroblasts, respectively [78,79]. The contribution of HIF in the genesis of PAH and vascular remodeling, was elegantly demonstrated by Dai and cols (2016) [71] through disruption of PHD2 activity in ECs and hematopoietic cells. These authors observed that ablation of *Egln1* (encoding PHD2) in ECs and hematopoietic cells (HCs; *Egln1Tie2*) in mice was sufficient to establish a phenotype strikingly different from the traditional models of chronic hypoxia (hypoxia- induced PH alone or hypoxia/Sugen 5416- induced PH) described by many authors [80,81,82,83]. These *Egln1 tie2 Cre* mice developed an irreversible obliterative vascular remodeling and pathophysiology similar to described in patients with severe PAH (idiopathic PAH), an effect that could be reverted using a HIF-2α translation inhibitor C76 (compound 76). Furthermore compound 76 also reversed PH induced with Sugen 5416/hypoxia and monocrotaline-induced in rat models PH [84].

A similar approach developed by Kapitsinou and cols (2016) [75] demonstrated that the PH response due to loss of endothelial PHD2 through genetic ablation of *Phd2* individually or in conjunction with either *Hif1a* or *Hif2a*, is dependent on HIF-2 but not HIF-1; these data also suggested that HIF-2 can regulate BMPR-II levels [75]. In concordance, HIF2α increases in lung vascular endothelial cells (LVECs) isolated from IPAH patients has been implicated in endothelial-to-mesenchymal transition associated with vascular lesions and remodeling [85].

It is suggested that one of the factors that may contribute to hypoxic pulmonary vasoconstriction that occur in PAH is the increase of arginase expression in endothelial cells stimulated by HIF 2α, triggering alterations in normal nitric oxide homeostasis [86]. As arginase is an indirect collagen synthesis precursor, possible decreases of NO bioavailability causes dysregulation of vascular tone through upregulation of adhesion molecules in the endothelium, which induces immune cell recruitment to the vascular wall, and stimulates SMC proliferation and migration [87], potentially due to increases arginine metabolite availability, such as polyamines [50]. HIF2α activation in ECs also up-regulates endothelin-1 (EDN1), a vasoconstrictor molecule, through signaling mechanisms that involve inhibition of the binding of vasodilator apelin (APLN) to its receptor (APLNR) [75].

The role of HIF-1α expression and activity during vascular remodeling in PAH has also been extensively studied. HIF 1α is increased in EC plexiform lesions in the lungs of IPAH patients [88,89]. In the metabolic context, both HIF-1 and HIF-2 contribute to altered metabolic phenotypes in PAECs by modulating the expression of distinct mitochondrial enzymes such as pyruvate dehydrogenase kinase 1 (PDK1), hexokinase 1,2 (HK1,2), lactate dehydrogenase A (LDHA), and glucose transporter 1,3 (GLUT1,3). All these enzymes regulate anaerobic glycolysis and the Warburg effect (aerobic glycolysis) during PH pathogenesis [90]. In SMCs, HIF-1α is decreased in PAH patients and myosin light chain phosphorylation (pMLC), a central determinant of vascular tone, is increased in patients with PAH, suggesting that in these cells HIF-1α works inversely to promote pulmonary vascular contractility [78]. Barnes and cols [78] discuss that the increases in HIF-1α found by other reports do not correspond to isolated studies of SMC, but in those associated with total lung tissue or ECs. In animal models, systemic loss of a single HIF-1α allele (Hif1a+/−) [91] or deficiency of HIF-1α in SMCs (HIF-1α -SMM-Cre) in mice exposed to hypoxia can cause lower right ventricular systolic pressure (RVSP) and less pulmonary vascular remodeling when compared to wild type hypoxic controls, indicating that HIF-1α inactivation in SMCs attenuates hypoxia-induced PH [77]. Among the various signaling stimuli promoted by HIF, one of the factors associated with pulmonary vasoconstriction is an increase in intracellular calcium concentration mediated by the transient receptor potential channel (TRPC). This assertion was based on the fact that PASMCs from hypoxia-exposed rats show an increased expression of TRPC1 and TRPC6 mRNA and proteins, and these channels were also increased in PASMCs from normoxic animals cultured under hypoxic conditions (4% O_2_; 60 h) [92], validating that the presence of HIF-1α in PASMCs is necessary for the development of PH. Interestingly, increases in endothelial calcium flux are linked to EC-SMC crosstalk, where inositol 3-phosphate (IP3) released by the SMC activates the IP3 receptor in EC in internal elastic lamina microdomains [93,94,95], called the myoendothelial junction (MEJ), triggering the stimulation of vasodilator pathways, such as the NO and endothelial-derived hyperpolarizing factor (EDHF) pathway [96,97,98,99,100]. Increases in calcium, mediated by IP3, will elicit endothelial vasoconstriction [101], which will contribute to vascular remodeling.

### 3.2. Cell Growth Factors

HIF-dependent genes regulate growth factors released by endothelium such as vascular endothelial growth factor (VEGF) (see [88]), epidermal growth factor (EGF), fibroblast growth factor 2 (FGF2) and platelet-derived growth factor (PDGF), which have a direct proliferative effect on neighboring PASMCs [102,103,104]. Similarly, the absence of HIF-1α in embryonic stem cells impairs cellular proliferation and VEGF expression during hypoxia [105]. In the hypoxia-induced PH model, PDGF activates the PI3Ks/AKT pathway resulting in cyclic adenosine monophosphate (cAMP) response element-binding protein depletion in PASMCs, which in turn induces switching of PASMCs from a contractile to a synthetic phenotype. This switch promotes cell proliferation, migration, and dedifferentiation, leading to pulmonary arterial remodeling [106]. PDGF-treated PASMCs exhibit significant increases in calpain activity, cell proliferation, and increased collagen-I protein level and this effect is abrogated in PASMCs lacking calpain-2 [107]. Postnatal deletion of PDGFR-β^+^/SMC marker^+^ progenitors completely prevent PH and right ventricle hypertrophy (RVH), attenuating pulmonary vascular remodeling in animal models of hypoxia induced PH [90,108]. Moreover, HIF-1α, HIF-2α or PDGF factor B (PDGF-B) knockout mice exposed to hypoxia have decreased PDGF-B mRNA accumulation in macrophages, and are protected against PH, RVH and pulmonary vascular muscularization [109].

**Figure 1:**
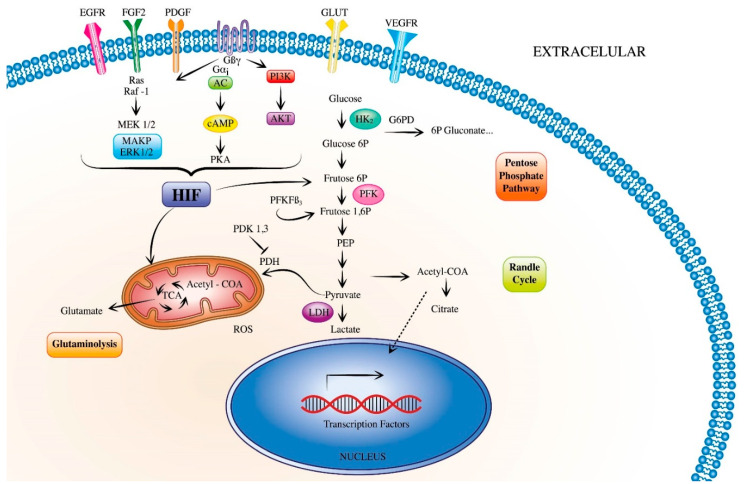
Metabolic signaling pathways promoting pulmonary hypertension. During the development of pulmonary hypertension, vascular cells release growth factors, which activate the hypoxia-inducible factors (HIF). HIF triggers signaling pathways that have a direct proliferative effect in both endothelial and smooth muscle cells. HIF can also modulate metabolic pathways such as glycolysis, pentose phosphate pathway, randle cycle, glutaminolysis and tricarboxylic acid cycle, which activate several downstream pathways. (EGFR, epidermal growth factor receptor; FGF2, fibroblast growth factor 2; PDGF, platelet-derived growth factor; GLUT, glucose transporter; HIF, hypoxia-inducible factors; ROS, reactive oxygen species).

Not only PDGF, but also Interleukin-6 (IL-6), endothelin-1 (ET1) and angiotensin II (Ang II) activate the signal transducer and activator of transcription-3 (STAT3), all of which are dysregulated in PAH patients. In a monocrotaline (MCT)-induced PH rat model a reciprocal relationship between loss of caveolin-1 (Cav-1) in PAECs and hyperactivation of phosphorylated STAT3 (pSTAT3) was found, and that was associated with an increase of proliferating cell nuclear antigen (PCNA) mRNA, indicating cell proliferation [35]. The inhibition of Janus kinase II (JAK2) impairs STAT3 activation in PAECs from PAH patients, reducing growth factors, decreasing proliferation and migration rates and promoting cell survival [36]. Furthermore, in PAECs of an ovine model of persistent PH of the newborn (PPHN), pSTAT3 binds to the eNOS promoter, decreasing its activity, thereby decreasing eNOS protein levels and NO production [37]. Together, these events contribute to migration, proliferation, and resistance to apoptosis in ECs and SMCs, which are hallmarks of vascular remodeling (Figure 1).

## 4. Role of Mitochondria in the Metabolic Reprogramming of PAH

Mitochondria are powerhouses that generate the majority of cellular energy but have other essential metabolic functions such as amino acid, lipid, and nucleotide synthesis, iron-sulfur cluster biogenesis, intermediate metabolite biogenesis and calcium homeostasis [110,111,112,113,114]. Importantly, mitochondrial signaling pathways can regulate innate immunity, programmed cell death as well as maintain cell survival and proliferation. These pleotropic functions make them essential to maintaining health of the organism. Thus, mitochondrial dysfunction is the primary cause of a number of metabolic diseases that are classified according to 3 types of genetic abnormalities, mitochondria DNA defects, nuclear DNA defects, or communication between mitochondria and nuclear DNA defects [115]. Multiple types of mitochondrial dysfunction have been identified in PAH and have contributed to the metabolic theory of PAH pathogenesis. However, as discussed below, activation or perturbation of any particular mitochondrial process, i.e., mitochondrial dynamics or bioenergetics, does not occur in isolation as modulation of these pathways may occur simultaneously or sequentially.

### 4.1. Mitochondria Dynamics

Mitochondria are arranged in dynamic networks that can rapidly undergo structural changes in response to changes in the cellular environment. These structural changes include mitochondrial division (fission), fusion and trafficking, a process termed mitochondrial dynamics [116]. Mitochondrial fission results in separation into small disconnected fragments while fusion produces larger interconnected networks that are connected to the endoplasmic reticulum. The balance between fission and fusion is a tightly controlled process and alteration of this balance affects mitochondrial quality control as well as cell apoptosis and proliferation. Mitochondrial dynamics are altered in PH since PASMCs isolated from PH patients have increased activation of dynamin related protein-1 (Drp1) and decreased expression of mitofusin-2 (Mfn2), hallmarks of mitochondrial fragmentation. Altered regulation of these proteins is associated with both increased mitochondria fragmentation and cell proliferation [117,118]. Hypoxic exposure of human PASMCs also increases Drp1 activation and mitochondrial fragmentation. Decreased membrane potential and respiration further indicated mitochondria dysfunction under these conditions. Pharmacological inhibition of Drp1 reversed the effects of hypoxia on mitochondrial structure, function and HPASMC proliferation [119].

ECs isolated from the Sugen/Hypoxia rat model also have increased mitochondrial fragmentation, proliferation, ROS, and intracellular calcium ([Ca^2+^]_i_) release via the transient receptor potential vanilloid-4 (TRPV4) calcium channel. Quenching ROS or inhibition of TRPV4 prevented mitochondrial fragmentation. Furthermore, quenching ROS prevented the [Ca^2+^]_i_ increase indicating a mechanistic link between ROS generation, calcium signaling and mitochondrial dynamics [120,121]. Although activation of fission and ROS generation often occur in conjunction, the mechanism by which ROS alters mitochondrial structure in pulmonary vascular cells is not completely elucidated [121,122,123]. In addition to coordinated signaling between ROS and mitochondrial dynamics, other mitochondrial processes, i.e., biogenesis and mitophagy, are interrelated with changes in mitochondrial dynamics.

### 4.2. Mitochondrial Biogenesis

Mitochondrial biogenesis is the process by which mitochondrial grow and divide to increase their mass, a process that requires coordination between fission and fusion. Mitochondria can autoreplicate their genome, which encodes the 13 subunits of the electron transport chain and the 22 tRNAs and 2rRNAs necessary for their translation. Over 1000 mitochondrial proteins are transcribed by nuclear DNA and imported into mitochondria to perform many mitochondrial functions. This process requires communication between the mitochondria and nucleus. Mitochondrial biogenesis is regulated by environmental stresses such as oxidative stress and cell division and proliferation as occurs in PH [124]. Dysregulated mitochondrial biogenesis is an early indicator of endothelial dysfunction, which is characterized by modified cell metabolism, reduced nitric oxide (NO) availability, and use [125,126,127,128] and increased oxidative stress. Generation of excess mitochondrial ROS disrupts endothelial nitric oxide synthase (eNOS) signaling, which further exacerbates ROS production and leads to dysfunctional mitochondria [129,130,131,132,133].

Both mitochondrial biogenesis and oxidative metabolism are regulated by peroxisome proliferator activated receptor *γ* coactivator-1 alpha (PGC-1α). Upregulation of PGC-1α attenuates oxidative damage [134] and promotes mitochondrial biogenesis, while downregulation induces mitochondrial dysfunction [135]. Hypoxia leads to perturbed endothelial cell function by decreasing PGC-1α expression causing mitochondria dysfunction manifested by decreased membrane potential and ATP production. Conversely, increasing PGC-1α expression mitigates the effects of hypoxia on the endothelium by decreasing ROS generation and inflammation, and by increasing eNOS activation and ATP production [136]. Furthermore, restoration of eNOS signaling using NO derivatives recovers PGC-1α expression and promotes mitochondria biogenesis [137,138]. Hence, defective mitochondria biogenesis disrupts multiple mitochondrial pathways in PAECs. However, defective mitochondria biogenesis also affects PASMCs function in PH. Decreased expression of PGC-1α leads to increased PASMC proliferation, fission and ROS generation, and decreased mitochondrial mass and impaired bioenergetics [139].

### 4.3. Mitophagy

Mitophagy is the process by which damaged mitochondria are degraded and removed via the autophagy pathway in order to maintain cell survival. Increased cellular levels of LC3B-I and LC3B-II are markers of autophagy activation [140]. Coordinated signaling between mitophagy and mitochondrial dynamics maintain mitochondria health and homeostasis. Mitochondria fragmentation, mediated by Drp1, is involved in quality control by promoting removal of damaged mitochondria. Conversely, MFN1 and MFN2 mediated fusion mitigates mitochondria stress by allowing healthy mitochondria to complement damaged mitochondria [141]. Mitophagy is involved in the pathogenesis of PH as fission is elevated in PASMCs isolated from PH patients, which suggests an impairment in the mitophagic clearance of dysfunctional mitochondria [142,143]. Furthermore, lungs from both IPAH and non-IPAH pulmonary hypertension patients have increased LC3B-I/II and autophagosomes [144]. However, the role of mitophagy in PH is not completely understood as mitophagy is detrimental or protective depending on the PH model, chronic hypoxia, Sugen/Hypoxia or MCT.

Mice exposed to chronic hypoxia have increased LC3B expression in both PAECs and PASMCs. Hypoxia also increases LC3B-I/II expression and proliferation in human PAECs and PASMCs. LC3B elevation under these conditions is part of a cellular attempt to mitigate PH as deletion of LC3B, in knockout mice or using siRNA in cells, leads to both decreased vascular resistance and vascular cell proliferation [145]. In rats exposed to chronic hypoxia, LCB3 levels are not elevated. However, when these hypoxic rats are treated with 17b-estradiol, LC3B-II levels increase and are associated with protection against vascular remodeling [146]. LC3B also mitigates vascular remodeling in rats exposed to Sugen/Hypoxia. While LC3B expression is increased in the pulmonary endothelium of these animals, treatment with rapamycin further enhances the expression but decreases PAECs proliferation [147]. In contrast to the chronic hypoxia models, MCT-induced PH in rats leads to increased LC3B expression that is not associated with protection against vascular remodeling. In this model, autophagy is inhibited by choloroquine treatment but rat PASMCs have higher apoptosis and decreased proliferation [148].

### 4.4. Calcium Signaling and Bioenergetics

Mitochondria have an important role as cellular oxygen sensors and changes in oxygen levels alter mitochondrial function and promote vasoconstriction of the pulmonary vasculature. Hypoxia alters mitochondrial ROS (mROS) generation, which leads to inhibition of voltage-gated potassium channels causing the cell membrane to depolarize which allows an influx of Ca^2+^ into the cytoplasm [118]. This increase in Ca^2+^ causes PASMCs to contract [147]. Chronic hypoxia also activates ER stress, which decreases the influx of Ca^2+^ from the ER to the mitochondria. This decrease in Ca^2+^ suppresses mitochondria function by inhibiting Ca^2+^ dependent enzymes such as PDH and α-ketogutarate (α-KG) but also decreases mROS, increases mitochondria membrane potential and suppresses apoptosis [148,149,150]. The effects of chronic hypoxia on mitochondrial enzymes leads to inhibition of glucose oxidation and efficient ATP production and promotes uncoupled glycolysis in the cytosol [17,117,151].

The UCP2 protein is a member of a family of 5 uncoupling proteins which are anion transporters located on the inner mitochondria membrane and functions to dissipate protons generated from the electron transport chain. However, UCP2 does not exhibit uncoupling activity in vascular mitochondria but functions as a Ca^2+^ channel and regulates Ca^2+^ influx from the endoplasmic reticulum to mitochondria [152,153,154]. PASMCs isolated from UCP2 deficient mice have hyperpolarized mitochondria, reduced activity of Ca^2+^ dependent mitochondrial enzymes and resistance to apoptosis [149].

PAECs from PH patients have decreased expression of UPC2 and increased markers of mitophagy [149]. Targeted knockout of UCP2 in ECs using cre-inducible floxed mice spontaneously induces PH that is worsened by intermittent hypoxia. Mitophagy also increased as determined by increased LC3B-II lipidation. siRNA deletion of UCP2 in mouse lung ECs exposed to chemical hypoxia also resulted in increased mitophagy and decreased mitochondrial biogenesis. Notably, mitochondria membrane potential was decreased, and apoptosis was increased [155]. Interestingly, PAEC apoptosis is proposed as an early event in PH development that potentially leads to emergence of apoptosis resistant ECs that are involved in vascular remodeling [156].

## 5. The Effects of Inflammatory Mediators on Vascular Remodeling

Although the current understanding of the impact of the immune response in PAH is still evolving, pathological studies have shown that a large number of inflammatory cells such as B lymphocytes, T lymphocytes, mast cells, and macrophages infiltrate the vessels of PAH patients [157]. However, how the vessel layers (intima, media and adventitia) act in conjunction to promote vascular changes in response to a variety of stimuli is not yet well defined. Adventitial cells can detect and direct responses to a wide variety of stimuli through paracrine communication with other adventitial cells or with cells from neighboring tissues. The production/expression of various cytokines/chemokines, their receptors and adhesion molecules by adventitial fibroblasts and recruited monocytes, has been documented as having a central role in PH vascular remodeling [43,157,158,159,160].

Some authors consider that IPAH is, in part, an autoimmune inflammatory disease, in which activated B cells in PAH can produce a variety of autoantibodies such as anti-endothelial cell antibodies. These antibodies accumulate in the intima of the pulmonary artery, which can induce endothelial cell apoptosis and adhesion molecule expression, leading to excessive proliferation of ECs and pulmonary vascular remodeling [161]. Recent studies have pointed out that cytotoxic T lymphocytes and helper T lymphocytes are found in PAH pulmonary artery plexiform lesions, and both play a role in promoting the process of vascular remodeling [162]. In contrast, regulatory T lymphocytes have immunosuppressive effects, which can regulate the inflammatory response within the pulmonary artery wall and inhibit vascular remodeling. In addition, mast cells participate in the pulmonary vascular remodeling of PAH by secreting chymotrypsin and tryptase. Tryptase can act on its target receptor and induce PAEC proliferation by activating the extracellular regulatory protein kinase signaling pathway, leading to changes in the structure and elasticity of the pulmonary artery [163].

Activated macrophages can also present antigens to T lymphocytes, leading to the activation of T lymphocytes and the production of T lymphocyte-mediated factors, thereby further promoting the occurrence and development of PAH-related inflammatory infiltration. In PAH patients there is an increased number of activated macrophages around the pulmonary blood vessels that could induce the release of IL-1, IL-6, IL-10, TNF-α, CCL2 (MCP-1) CXCL1 (fractalkine) and CCL5 (RANTES) [157,164,165]. A schematic mechanism of vascular remodeling led by IL-6 overexpression was proposed by Furaya and cols (2010) [166] and Steiner and cols (2009) [167] using animal models. These authors hypothesized that proliferation and resistance to apoptosis in SMC is a consequence of IL-6-stimulated VEGF upregulation, which also induces BMPR2 and TGFβR downregulation. In ECs, exposure to IL-6 triggers apoptosis through the inhibition of Tie2 to a mechanism that disrupts Ang-1 expression in SMC. This stimulus induces the recruitment of inflammatory cells, such as lymphocytes and monocytes, further increasing the production of cytokines and IL-6 by ECs and SMCs. Furthermore, in patients and PAH experimental models, epigenetic changes stimulate and induce the proliferation of vascular fibroblasts, and this change is related to the activation of macrophages and the activation of a large number of pro-inflammatory cytokines mediated by HDAC1 [168].

Pro-inflammatory macrophages stimulate hexokinase 1 expression by upregulating NLRP3 inflammasome activation [169]. Inflammasome activation consists of a series of chain reactions starting with caspase-1 activation and promoting pro-IL-18 and pro-IL-1 maturation. Although it is still controversial whether glycolysis favors positively or negatively the activity of NLRP3 inflammasome. Not only hexokinase, but also PKM2 and other glycolytic regulators have been associated with NLRP3 inflammasome activity [169,170,171,172]. It has been seen that during vascular endothelial dysfunction, ECs promotes activation of programmed cell death through upregulation of caspase-1 [173]. Damage signal molecules released by PAEC can stimulate the production of IL-1β, and the combination of IL-1β and IL-1R1 can lead to the activation of NF-κB and the synthesis of IL-6 and TNF-α, which stimulate PASMC proliferation [174]. Studies have shown that after IL-1R1 knockout, IL-1β-mediated PASMC proliferation is inhibited [175] and that IL-1 antagonists can effectively reduce vascular inflammation while significantly slowing pulmonary vascular remodeling [176].

**Figure 2:**
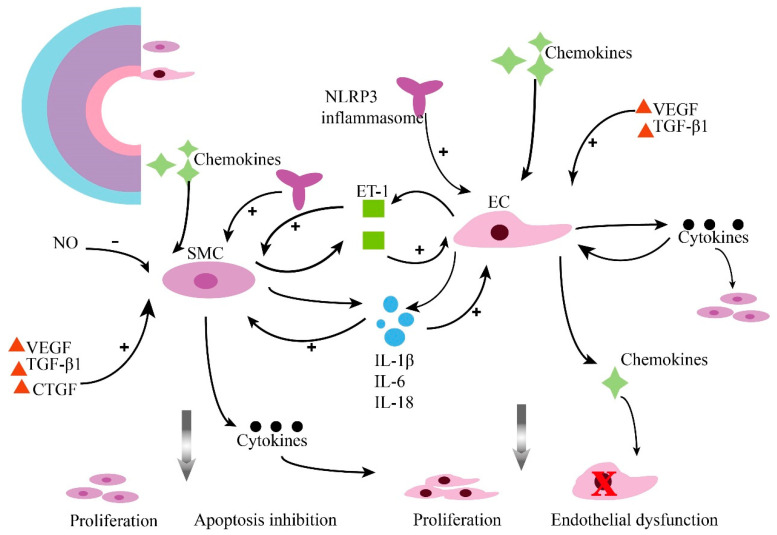
Inflammation contributes to pulmonary vascular remodeling in PAH. Pulmonary vascular lesions subsist in a tremendously inflammatory microenvironment. Pulmonary arterial smooth muscle cells (PASMCs) and pulmonary arterial endothelial cells (PAECs) crosstalk, resulting in changed sensitivity to inflammatory factors and their promoted potential to promote the inflammatory response. (ET-1, endothelin 1; SMC, smooth muscle cells; EC, endothelial cells; NO, nitric oxide; VEGF, vascular endothelial growth factor; TGF-β1, transforming growth factor-β1; IL-1β, interleukin 1-beta; IL-6, interleukin 6; IL-18, interleukin-18; CTGF, connective tissue growth factor; NLRP3, Nod-like receptor protein 3).

It is increasingly clear that in the vascular microenvironment of PAH, glycolysis, fatty acid oxidation and the production of reactive oxygen species involved in metabolic changes are affected by a variety of complex interactions, not only between ECs and SMCs, but also fibroblasts and macrophages. These studies show that bridging the research of metabolism and inflammation will help us to understand the pathogenesis of PAH more deeply and comprehensively, and the relationship between dysregulated immune response and metabolic changes in PAH will be an important goal in future research (see Figure 2).

## 6. Clinical Trials Targeting Metabolic and Inflammatory Signaling in PAH

### 6.1. Animal Models of PH

Preclinical rodent models of PH have been an essential tool used to identify pathways and molecules to target for PAH therapeutic intervention. The three major models used to induce PH in rodents are hypoxia induced PH (HPH), Sugen-hypoxia (SuHyp) and monocrotaline (MCT). These models result in mild (i.e., HPH) to severe (i.e., MCT or SuHyp) PH and display different disease characteristics based on the type of rodent, which has been extensively described by other groups [177,178]. Importantly, because of the heterogeneity of the human disease, no one model completely recapitulates the disease characteristics observed in patients. Nevertheless, these preclinical models are a critical means to discovering novel mechanisms to treat PAH and have led to clinical trials involving drugs that target multiple signaling mediators and cellular processes.

### 6.2. Standard PAH Interventions

Currently, therapies for PAH are categorized into four classes of drugs that were originally developed as vasodilators: (1) prostacyclin synthetics and analogs, (2) endothelin receptor antagonists (ERAs), (3) phosphodiesterase type 5 inhibitors (PDE5i) and (4) soluble guanylate cyclase (sGC) stimulators [179]. Unfortunately, none of these drugs can effectively reverse the disease, and in many cases, lung or lung and heart transplantation may be the only life-saving option for the many patients with poor prognosis. In addition, these therapies are not selective for the pulmonary circulation, and, whereas some were subsequently shown to limit the proliferative potential of vascular cells, they do not act directly on mechanisms of hyperproliferative and apoptosis-resistant remodeling of the pulmonary arteries [179].

### 6.3. Current Clinical Trials

Because PAH is a complex disease that involves signaling of many pathways and signaling mediators, interventions have targeted multiple pathways, which have been extensively reviewed by Sommer and cols. [3]. Studies involving compounds such as dichloroacetate (DCA), which normalizes glucose oxidation, inhibiting pyruvate dehydrogenase kinase, and trimetazidine and ranolazine, which act by modulating the balance between fatty acid and glucose oxidation, have shown promise in the treatment of PAH [45,180]. For instance, in a small trial (ClinicalTrials.gov identifier NCT01083524) dichloroacetate treatment improved hemodynamics and functional capacity in genetically susceptible patients with PAH, without clinically significant change in QT intervals, cardiac rhythm, and liver, bone marrow, and renal functions [45]. However, all patients did not respond to DCA, likely due to it is rapid clearance and small therapeutic window. Still, these studies were an important step in suggesting metabolic reprogramming as a fundamental feature of the molecular pathogenesis of PAH and reinforces the importance of understanding metabolic disturbances in disease initiation, maintenance, and mitigation via the use of therapeutic interventions. While there are multiple clinical trials underway to target various cellular processes, herein we have summarized the trials that specifically involve drugs that target metabolic and inflammatory pathways (Table 1).

## 7. Conclusions

Several events contribute to the pathogenesis of PAH and pulmonary vascular remodeling. It is clear, however, that metabolic and inflammatory dysregulation are central to the pathogenesis of PAH. Questions remain whether these disturbances play a causal or a compensatory (mal)adaptive role in the disease. There are still multiple opportunities to better understand the role of pulmonary vascular EC-SMC behavior in the maintenance of vascular homeostasis in the response to injury and in the development of pulmonary vascular remodeling. Finally, the therapeutic role of strategies counteracting metabolic and inflammatory dysregulation in PAH needs to be established.

## Figures and Tables

**Table 1:** Current PAH Clinical Trials Targeting Metabolic and Inflammation Pathways.

Pathway	Drug name and ClinicalTrials.gov ID	Targeted Pathway or Process	Trial Phase	Reference or Status
Metabolic	Apabetalone (APPRoAch-p): NCT03655704	Bromodomain-containing protein-4: Cell growth, vascular remodeling	Phase 2	Currently recruiting
	CXA-10 (PRIMEx-nitrated fatty acid compound): NCT03449524	NF-kB inhibitor, Nrf2 Activator: inflammation, antioxidant	Phase 2	Terminated 2020-no results reported
	Ferinject or CosmoFer: NCT01447628	Iron Infusion	Phase 2	Howard et al. *Ann. Am. Thorac. Soc.* June 2021 [181]
	Metformin (biguanide): NCT03617458	Insulin Resistance	Phase 2	Currently recruiting
	Olaparib (OPTION): NCT03782818	PARP inhibitor: DNA damage	Phase 1	Currently recruiting
	Ranolazine: NCT01839110 and NCT02829034	Sodium channel inhibitor, fatty acid oxidation inhibitor	Phase 4	Han et al. *J. Card Fail.* Feb 2021 [182]
	Trimetazidine: NCT02102672	Fatty acid oxidation inhibitor	Phase 2	2 years post completion date: Status Unknown
Inflammation	Rituximab: NCT01086540	anti-CD20 antibody, targets B-lymphocytes	Phase 2	Zamanian et al. *Am. J. Respir. Crit. Care Med.* July 2021 [183]
	Elafin: NCT03522935	Inhibits neutrophil serine proteases elastase and proteinase-3	Phase 1	Completed April 2021: No results posted
Metabolic/Inflammation	Bardoxolone methyl: NCT02036970	IkB Kinase and NF-kB Inhibitor, Nrf2 Activator: inflammation, antioxidant	Phase 2	Results Posted 23 July 2021
	Bardoxolone (CATALYST): NCT02657356	IkB Kinase and NF-kB Inhibitor, Nrf2 Activator: inflammation, antioxidant	Phase 3	Terminated due to safety risk during COVID-19 Pandemic
	Bardoxolone (RANGER): NCT03068130	IkB Kinase and NF-kB Inhibitor, Nrf2 Activator: inflammation, antioxidant	Phase 3-Long term safety study	Terminated due to safety risk during COVID-19 Pandemic
	ABI-009 (albumin bound rapamycin): NCT02587325	mTOR inhibitor, HIF signaling: immunosuppressant, cell growth and motility	Phase 1	Currently recruiting

## Abbreviations

[Ca^2+^]_i_	intracellular calcium concentration
Ang II	angiotensin II
BMPR2	Bone Morphogenetic Protein Receptor Type 2
BOECs	blood outgrowth endothelial cells
cAMP	cyclic adenosine monophosphate
Cav-1	caveolin-1
Drp1	dynamin related protein-1
ECs	endothelial cells
EGF	epidermal growth factor
ENO	enolase
eNOS	endothelial nitric oxide synthase (eNOS)
ERAs	endothelin receptor antagonists
ERK	extracellular signal-regulated kinase
ET1	endothelin-1
FGF2	fibroblast growth factor 2
G6PD	glucose-6-phosphate dehydrogenase
GLUT	glucose transporter
HIF1	hypoxia-inducible factors 1
HIF2	hypoxia-inducible factors 2
HK1,2	hexokinase 1,2
HLMVECs	human lung microvascular endothelial cells
HPAH	hereditary pulmonary arterial hypertension
HREs	hypoxia-responsive elements
IL18	interleukin 18
IL-6	interleukin-6
IPAH	idiopathic pulmonary arterial hypertension
JAK2	Janus kinase II
LDHA	lactate dehydrogenase A
MCT	monocrotaline
Mfn2	mitofusin-2
MIF	migration inhibitory factor
miRNAs	microRNA
MMP	metalloproteinases
mPAP	mean pulmonary artery pressure
mROS	mitochondrial ROS
NADPH	nicotinamide adenine dinucleotide phosphate
NF-κB	nuclear factor-kappa B
NO	nitric oxide
OXPHOS	oxidative phosphorylation
PAECs	pulmonary artery endothelial cell
PAH	pulmonary arterial hypertension
PASMCs	pulmonary artery smooth muscle cells
PCNA	proliferating cell nuclear antigen
PDE5i	phosphodiesterase type 5 inhibitors
PDGF	platelet-derived growth factor
PDH	pyruvate dehydrogenase
PDK	pyruvate dehydrogenase kinase
PFKFB3	6-phosphofructo-2-kinase/fructose-2,6- bisphosphatase 3
PGC-1α	peroxisome proliferator activated receptor gamma coactivator 1-alpha
PGI2	prostaglandin 2
PH	pulmonary hypertension
PKM	pyruvate kinase muscle
PPHN	persistent pulmonary hypertension of the newborn
PPP	pentose phosphate pathway
PTBP1	polypyrimidine tract binding protein 1
PVR	pulmonary vascular resistance
REVEAL	registry to evaluate early and long-term disease management in PAH
ROS	reactive oxygen species
RVH	right ventricle hypertrophy
RVSP	right ventricular systolic pressure
sGC	soluble guanylate cyclase
SIRT3	sirtuin 3
SMCs	smooth muscle cells
SOD	superoxide dismutase
STAT3	signal transducers and activators of transcription-3
TCA	tricarboxylic acid cycle
TGF-β1	transforming growth factor-β1
TIMP-1	tissue inhibitors of metalloproteinases-1
TNF-α	tumor necrosis factor alpha
TRPC	transient receptor potential
TRPV4	transient receptor potential vanilloid-4
UCP2	uncoupling protein 2
VEGF	vascular endothelial growth factor
VSMCs	vascular smooth muscle cells
α-KG	α-ketoglutarate
